# Closing the medullary canal after retrograde nail removal using a bioabsorbable bone plug: technical tip

**DOI:** 10.1007/s00402-012-1483-x

**Published:** 2012-02-17

**Authors:** T. Schepers, L. M. M. Vogels

**Affiliations:** Department of Surgery-Traumatology, Erasmus MC, University Medical Center Rotterdam, Room H822-k, P.O. Box 2040, 3000 CA Rotterdam, The Netherlands

**Keywords:** Implant removal, Retrograde nail, Hemarthrosis knee, Bone plug

## Abstract

We describe a simple technique for closure of the intra-articular opening after the removal of a retrograde femur nail. With the use of a gelatine bioabsorbable bone plug the medullary canal is closed, reducing leakage of blood and cancellous bone particles from the bone into the knee joint.

## Technical note

Retrograde intramedullary nailing is a frequently used technique in distal femoral fractures [3, 5]. Normally there is no need to remove this nail; however, should there be an indication for removal of the implant, an open connection between the medullary canal and the knee joint remains. Subsequently, most patients experience hemarthrosis of the knee joint to some extent. This hematoma, which likely contains small cancellous bone particles with pluripotent stem cells from the medullary canal, has an arthritis-like effect intra-articular in the knee joint [2, 6, 7].

**Fig. 1:**
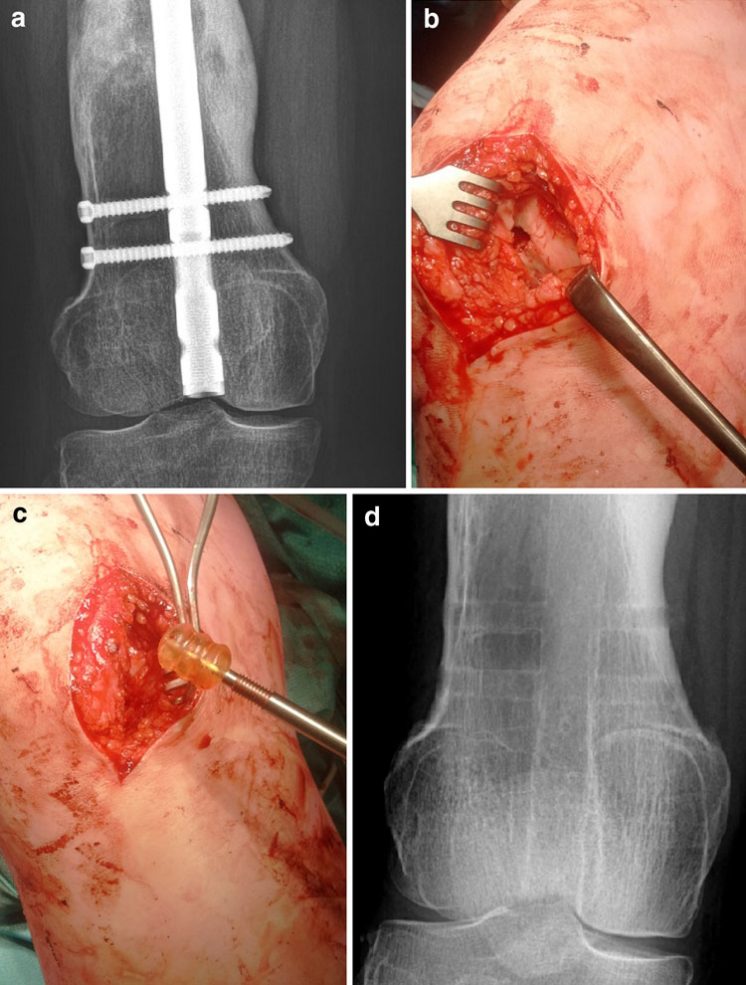
**a** Pre-operative image of distal femur with a 12 mm intra-medullary nail in place **b** Intra-operative image showing the opening to the medullary canal following removal the nail **c** Placement of an 11 mm gelatine bioabsorbable bone plug into the opening of the medullary canal **d** Post-operative image

In patients suffering from hemophilia or those who need an early restart of anticoagulant treatment, there might be an increase in drainage from the medullary canal with concomitantly knee hemarthrosis [4, 9]. We have encountered several cases with prolonged hemarthrosis of the knee, even after use of a wound drain [1]. In cases where we pre-operatively expect or peroperatively observe an increase in drainage from the intramedullary canal, we now place a bioabsorbable bone plug in the entry site of the retrograde nail (Fig. 1). This plug is similar as the one used prior to cement insertion in the proximal femur for the placement of a (hemi-)arthroplasty of the hip. This plug is made from animal pharmaceutical gelatine, purified water, and glycerol, and absorbs in a few weeks [8].

Choosing the appropriate size of the plug can be achieved in two different ways: (1) using the diameter of the removed nail, (2) using the measuring probes of the (hemi-) arthroplasty set. One millimetre is usually subtracted from the measured size or from the nail diameter to facilitate an easier placement. The plug is placed approximately 2 cm into the canal and remains firmly in place by the absorption of water. We have thus far not encountered problems of a dislodging bioresorbable plug.

The gelatine plug is advantageous over the use of bone wax as plug, because bone wax does not dissolve over time and might dislodge into the joint. Secondly, bone wax has been frequently the cause of a reactive foreign-body granuloma or even allergic reactions [8].

In conclusion, upon removal of a retrograde intramedullary nail from the femur the open connection of the knee joint and medullary canal can be closed easily using a gelatine bone plug. This technique might also be applicable in other regions, for example after the removal of an antegrade femoral or tibial nail, to prevent hematoma in the surrounding soft-tissues.

## References

[1] Crevoisier XM, Reber P, Noesberger B (1998). Is suction drainage necessary after total joint arthroplasty? A prospective study. Arch Orthop Trauma Surg.

[2] Horisberger M, Kazemkhani S, Monument MJ, Emmenegger D, Hildebrand KA, Herzog W (2011) Does the source of hemarthrosis influence posttraumatic joint contracture and biomechanical properties of the joint? Clin Biomech (Bristol, Avon) 26(7):790–795 10.1016/j.clinbiomech.2011.02.013 21420211

[3] Lucas SE, Seligson D, Henry SL (1993). Intramedullary supracondylar nailing of femoral fractures A preliminary report of the GSH supracondylar nail. Clin Orthop Relat Res.

[4] Mortazavi SM, Heidari P (2008). Retrograde intramedullary nailing of supracondylar femoral fractures in haemophilic patients. Haemophilia.

[5] Papadokostakis G, Papakostidis C, Dimitriou R, Giannoudis PV (2005). The role and efficacy of retrograding nailing for the treatment of diaphyseal and distal femoral fractures: a systematic review of the literature. Injury.

[6] Pforringer W (1982). Hemarthrosis and cruciate ligaments—biomechanical studies. 1. Unfallchirurgie.

[7] Safran MR, Johnston-Jones K, Kabo JM, Meals RA (1994). The effect of experimental hemarthrosis on joint stiffness and synovial histology in a rabbit model. Clin Orthop Relat Res.

[8] Schonauer C, Tessitore E, Barbagallo G, Albanese V, Moraci A (2004). The use of local agents: bone wax, gelatin, collagen, oxidized cellulose. Eur Spine J.

[9] Solimeno L, Goddard N, Pasta G, Mohanty S, Mortazavi S, Pacheco L, Sohail T, Luck J (2010). Management of arthrofibrosis in haemophilic arthropathy. Haemophilia.

